# Isthmus Stenosis or Co-Arctation of the Aorta

**Published:** 1909-08-21

**Authors:** 


### DISEASES OF CHILDREN.

### ISTHMUS STENOSIS OR CO-ARCTATION OF THE AORTA.

SOME of the rarer forms of congenital mal-development of the heart or circulatory organs deserve consideration for the simple reason that they are present at birth, and frequently diagnosable then or in the first few months of life. The peculiar malformation formerly known as Co-arctation of the Aorta, but in more recent years better named Isthmus Stenosis, comes under this description. In 1900 there were over 100 cases on record, divisible among males and females in the proportions of 7 to 2. The isthmus of the aorta is that portion of the aortic root between the fourth and fifth branchial arches, that is, the portion between the origin of the subclavian artery and the entrance of the ductus Botalli into the aorta. During fœtal life very little circulatory strain falls on this portion of the aorta, and it remains thin and narrow. After birth it develops rapidly. The whole of this part of the aorta may be absent; or it may be reduced to a fibrous cord for about one centimetre; or there may be a double constriction, with a dilated intervening section; or it may be pervious, but the lumen as small as that of a No. 1 catheter. If it is pervious the condition is spoken of as Incomplete Co-arctation. The majority of cases are of this type. Only about 15 of the complete variety are on record, and sometimes the term co-arctation is limited to the complete variety.

The ductus arteriosus may supply or give rise to the descending aorta, owing to this failure of development. If the isthmus aortæ is absent, the transverse and descending aorta are completely separated. In still rarer cases the ductus is also absent. The constriction of the isthmus may be sudden or gradual. The vessel is usually dilated or funnel-shaped on the proximal side, and the artery beyond is thin and small in calibre.

If the ductus is absent in these cases, the collateral circulation is carried on by the anastomoses between the upper intercostal arteries from the second part of the subclavian with the first aortic intercostals; and by those of the posterior scapular arteries from the second part of the subclavian, the subscapular branches, and the internal mammary arteries. Most commonly the internal mammary arteries are the most dilated, forming tortuous pulsating cords under the skin, and, together with the anterior and superior intercostal, subscapular, scapular and external thoracic arteries, anastomose with the epigastric and the posterior intercostal arteries.

Cases of mild grade develop no collateral circulation, but the left ventricle is hypertrophied and the aortic second sound accentuated. The ventricle, in more serious cases, eventually fails to stand the strain of the anastomotic circulation, and death results from cardiac failure.

Isthmus stenosis is often associated with valvular and arterial anomalies, and other congenital malformations—*e.g.* patent ductus; the innominate and common carotid, or the left subclavian and common carotid, arising from a single trunk; irregular origin of the vertebral arteries; transposition of the heart; patency of the undefended space; secondary aortic valve disease, or only two aortic cusps; or defects such as hare-lip, cleft palate, hypospadias, and deformity of the lower limbs. The supply of blood to the upper limbs may be quite normal; and the stenosis limited to that part of the aorta immediately above the ductus or just where the ductus enters.

The effects on the circulation develop gradually, and the lesion, if slight, will not be diagnosed in early life. Later on, it gives rise to many thickened and tortuous arteries under the skin; excessive pulsation in the arteries of the head, neck and upper extremities; exaggerated radial pulses; murmurs in the arteries of the neck, upper intercostal spaces, and all over the precordium. The murmur is loudest over the manubrium. The aorta bulges forward in the neck, and there may be dulness, thrill and pulsation in the second right interspace. The pulse in the abdominal aorta and the femorals, etc., is feeble or absent. If the stenosis is incomplete, a systolic murmur may be heard over the descending aorta between the scapula and the spine. Schlesinger described congenital stenosis of the abdominal aorta in a woman aged 31, in whom a loud whirring systolic murmur was heard from the sixth dorsal vertebra downwards into the femoral arteries, the pulse being weak in the femorals and hardly felt in the arteries of the foot. This was possibly an instance of isthmus stenosis. In a girl, aged five weeks, under the writer's care there was no cardiac murmur and no lividity, except in occasional attacks and on crying. Death was due to capillary bronchitis. The aorta gave off normal vessels and then abruptly narrowed at the isthmus. The ductus was patent and much dilated, so that the aorta seemed a normal continuation of it; and the ventricular septum was patent in the usual situation. The right ventricle was thick-walled and the left was both small and thin-walled. This was a simple case of stenosis which was compatible with life, had it not been for the attack of bronchitis. Some of these patients may live a long time. One of Raynaud's lived to 92. The average duration of life is 30 years, possibly much less, for cases in very early life are apt to be overlooked and not included. Symptoms may be slight or absent, perhaps limited to dyspnœa on exertion, unless there is secondary cardiac failure. In a navvy under the writer's observation many years ago there were no symptoms whatever, but the arterial dilatation and tortuosity were very marked.

They are particularly interesting on account of the difficulty in diagnosis and prognosis. It may be stated in general terms that the prognosis varies inversely as the degree of stenosis, and directly as the completeness of the collateral circulation. If there is only a moderate degree of narrowing, enough to render the pulsation in the arteries of the lower limbs a little feebler than those of the upper limbs, it is likely that life may be prolonged to the usual term.

